# C/EBPα and C/EBPβ Are Required for Sebocyte Differentiation and Stratified Squamous Differentiation in Adult Mouse Skin

**DOI:** 10.1371/journal.pone.0009837

**Published:** 2010-03-23

**Authors:** John S. House, Songyun Zhu, Rakesh Ranjan, Keith Linder, Robert C. Smart

**Affiliations:** 1 Cell Signaling and Cancer Group, Department of Environmental and Molecular Toxicology, North Carolina State University, Raleigh, North Carolina, United States of America; 2 Department of Population Health and Pathobiology, College of Veterinary Medicine, North Carolina State University, Raleigh, North Carolina, United States of America; Texas Tech University Health Sciences Center, United States of America

## Abstract

C/EBPα and C/EBPβ are bZIP transcription factors that are highly expressed in the interfollicular epidermis and sebaceous glands of skin and yet germ line deletion of either family member alone has only mild or no effect on keratinocyte biology and their role in sebocyte biology has never been examined. To address possible functional redundancies and reveal functional roles of C/EBPα and C/EBPβ in postnatal skin, mouse models were developed in which either family member could be acutely ablated alone or together in the epidermis and sebaceous glands of adult mice. Acute removal of either C/EBPα or C/EBPβ alone in adult mouse skin revealed modest to no discernable changes in epidermis or sebaceous glands. In contrast, co-ablation of C/EBPα and C/EBPβ in postnatal epidermis resulted in disruption of stratified squamous differentiation characterized by hyperproliferation of basal and suprabasal keratinocytes and a defective basal to spinous keratinocyte transition involving an expanded basal compartment and a diminished and delayed spinous compartment. Acute co-ablation of C/EBPα and C/EBPβ in sebaceous glands resulted in severe morphological defects, and sebocyte differentiation was blocked as determined by lack of sebum production and reduced expression of stearoyl-CoA desaturase (SCD3) and melanocortin 5 receptor (MC5R), two markers of terminal sebocyte differentiation. Specialized sebocytes of Meibomian glands and preputial glands were also affected. Our results indicate that in adult mouse skin, C/EBPα and C/EBPβ are critically involved in regulating sebocyte differentiation and epidermal homeostasis involving the basal to spinous keratinocyte transition and basal cell cycle withdrawal.

## Introduction

There are six members of the CCAAT/enhancer binding protein (C/EBP) family: C/EBPα, C/EBPβ, C/EBPγ, C/EBPδ, C/EBPε and C/EBPζ. C/EBPs are members of the basic leucine zipper (bZIP) class of transcription factors. C/EBPs play important roles in fundamental cellular processes including proliferation, apoptosis, differentiation, inflammation, senescence and energy metabolism [1], [2]. In terms of differentiation, C/EBPα plays a role in the differentiation of myeloid cells [3], [4], alveolar type II cells [5], hepatocytes [6], [7] and adipocytes [8]–[10] while C/EBPβ has a role in the differentiation process in mammary epithelial cells [11], [12], adipocytes [13], ovarian granulosa cells [14], and macrophages [15], [16].

The interfollicular epidermis (IFE) of skin is a stratified squamous epithelium composed primarily of keratinocytes that undergo a highly coordinated program of sequential changes in gene expression as they migrate from the proliferating basal keratinocyte layer through morphologically distinct spinous and then granular suprabasal keratinocyte layers, ending in the production of a nonviable stratum corneum. A discrete population of IFE stem cells replenishes the basal cell compartment in the epidermis[17]. Epidermal homeostasis involves the intricate regulation and balance between proliferation of basal cells and their commitment to terminally differentiate. Notch signaling [18]–[20] and AP-2α/AP-2γ [21] have essential roles in epidermal development and postnatal epidermal differentiation.

C/EBPα and C/EBPβ have been shown to be highly expressed in mouse and human epidermis [22]–[24] suggesting a possible role for these transcription factors in stratified squamous differentiation. However, the analysis of an epidermal specific germline C/EBPα knockout mouse revealed that C/EBPα is completely dispensable for epidermal homeostasis involving squamous differentiation and keratinocyte proliferation [25] and the analysis of germline C/EBPβ knockout mouse epidermis revealed changes, albeit modest, in keratinocyte differentiation involving decreased K1 and K10 expression and increased basal keratinocytes proliferation, indicating a role for C/EBPβ in the early events of stratified differentiation [26]. Because these changes in C/EBPβ deficient epidermis were modest it led to the notion that C/EBPβ has supportive rather than a major role in epidermal keratinocyte differentiation. However, another possibility for the lack of a major skin phenotype in the C/EBPα and C/EBPβ knockout mouse is that functional redundancies between the transcription factors mask their roles in the single knockouts. In support of this notion are studies demonstrating that the knockin of C/EBPβ into the C/EBPα locus can rescue C/EBPα^−/−^ mice from perinatal lethality [27] and restore granulocytic differentiation and glycogen metabolism [27]. A recent study in developing mouse skin in which C/EBPα and C/EBPβ were co-deleted during epidermal development produced mice that were only viable for a few hours after birth, and the analysis of epidermis from these mice revealed hyperplasia and decreased expression of spinous and granular markers of differentiation [28]. Thus, while C/EBPα and C/EBPβ are critical for epidermal development, their roles in stratified squamous differentiation in the adult epidermis remain poorly understood.

Sebaceous glands extend off of the upper hair follicle and are formed from a sebocyte progenitor that expresses Blimp 1, keratin 14 (K14) and keratin 5 (K5) [29] and these cells produce a proliferative population of sebocytes which in turn differentiate to produce the lipid/sebum producing cells [30]. These differentiated sebocytes lyse and release their contents of lipids/sebum into the hair follicle canal and make their way to surface of the skin where they prevent drying of the skin and hair [31]. cMyc [30], [32], hedgehog signaling [33] and AP-2α/AP-2γ [21] regulate sebaceous gland development/differentiation. C/EBPα and C/EBPβ are abundantly expressed in sebocytes of the sebaceous glands [34], [35], however, no function for these transcription factors in sebocytes has been described and no sebaceous gland phenotype was reported in C/EBPα or C/EBPβ knockout mice.

To address possible functional redundancies and to reveal the functional roles of C/EBPα and C/EBPβ in the adult epidermis and sebaceous gland, mouse models were developed in which either family member could be ablated alone or together in the epidermis and sebaceous glands of adult mice. Our results demonstrate C/EBPα and C/EBPβ are essential for sebocyte differentiation and epidermal squamous differentiation involving the basal to spinous transition.

## Methods

### Ethics statement

All animal work described in the study involving animal husbandry, experimentation, and care/welfare have been conducted according to NIH guidelines and approved by NCSU Institutional IACUC committee.

### Animals and treatments

K14-CreER^tam^ homozygous mice [36] (CD1) were obtained from Jackson Labs (#005107) and crossed with either floxed-α (B6.129)[37], floxed-β (B6.129)[38] or floxed-αβ (B6.129). Floxed-α and floxed-β mice were genotyped as previously described [37], [38]. Mice were genotyped for K14-CreER^tam^ using the following primers: forward, CGATGCAACGAGTGATGAGGTTC; reverse, GCACGTTCACCGGCATCAAC. Mice 6–8 weeks of age in telogen were treated topically to clipped skin with 4OHT (Sigma catalog # T176-50MG) 1.0 mg/day for days 1–5 and again on days 13–17 dissolved in 95% ethanol (1.0 mg/200 ul) and skin collected for IHC on day 21.

### Preparation of epidermal homogenates

Mice were killed by cervical dislocation and dorsal skin was removed and subjected to 15 seconds heat shock in 65°C deionized water followed by 15 seconds in ice water. Water was removed from skin by blotting between paper towels and epidermis was scraped from dermis. For C/EBPα and C/EBPβ proteins, epidermal scrapes were homogenized on ice in RIPA buffer (1.0% NP-40, 0.5% sodium deoxycholate, 0.1% SDS, 1.5 mM sodium orthovanadate, 1.5 mM phenylmethylsufonyl fluoride, 1.5 mM dithiothreitol, and 1x protease inhibitor cocktail (Roche Diagnostics GmbH ref 11 836 001) in PBS), and centrifugated at 14,000 g for 20 minutes at 4°C. Supernatants were stored at −80°C prior to use. Protein quantification was determined by Bio-Rad Protein Assay reagent (Bio-Rad, Cat# 500–0006). For keratins and cornified envelope precursors, epidermal scrapes were homogenized in 20% 2-mercaptoethanol and 5% SDS, boiled 5 minutes, allowed to cool and centrifugated at 14,000 g for 10 minutes.

### Immunoblot analysis

Equal amounts of protein were denatured in sample buffer, loaded onto 12% tris-glycine gels (Invitrogen, EC6005), separated by gel electrophoresis and transferred to PVDF membrane. Membranes were blocked for one hour (PBS with 0.1% tween/5.0% milk/1.0% BSA), and incubated overnight at 4°C (PBS with 0.1% tween/1.0% BSA) with one of the following rabbit polyclonal antibodies: C/EBPα (Santa Cruz, SC-61, 1∶2000), C/EBPβ (Santa Cruz, SC-150, 1∶2000), K1 (Covance, 1∶10,000), K5 (Covance, 1∶10,000), K10 (Covance, 1∶10,000), K14 (Covance, 1∶10,000), involucrin (Covance, 1∶10,000), loricrin (Covance, 1∶10,000), filaggrin (Covance, 1∶10,000). Membranes were then rinsed one hour in PBS-T (0.1% tween) and subjected to one hour incubation (PBS-T, 1.0% BSA) with anti-rabbit IgG, horseradish peroxidase-linked secondary (GE Healthcare, NA934V, 1∶2500), incubated one minute with Western Lightening Plus-ECL (PerkinElmer NEL105001EA) and subsequently exposed to film. Each blot represents n≥3 mice per genotype. Membranes were stripped and re-probed for β-actin (Sigma A-5441, 1∶25,000, mouse) for one hour followed by one hour incubation anti-mouse horseradish peroxidase-linked secondary (GE Healthcare, NXA931, 1∶25,000), incubated one minute with Western Lightening Plus-ECL (PerkinElmer NEL105001EA) and subsequently exposed to film.

### Cell proliferation analysis

Mice were injected intraperitoneally with bromodeoxyuridine (BrdU, Sigma B5002, 100 mg/kg in PBS) and killed 1 hour later. Skin was fixed in 10% neutral buffered formalin phosphate (NBF) for 20 h and moved to 70% ethanol and subsequently embedded in paraffin. Immunohistochemical (IHC) staining for BrdU was performed as previously described [23] on 5 µM sections with anti-BrdU (BD Biosciences 27644, 1∶25).

### Immunohistochemical (IHC) staining

Tissues were fixed in 10% NBF for 20 h, switched to 70% ethanol and embedded in paraffin. Tissue sections (5 µM) were stained for H&E or specific IHC as described [23], [26], [39] with the following antibodies: C/EBPα (Santa-Cruz SC-61, 1∶1000), C/EBPβ (Santa-Cruz SC-7962, 1∶1000), K5 (Covance PRB-160P, 1∶2000), K10 (Covance PRB-159P, 1∶2000), involucrin (Covance PRB-140C, 1∶2000), loricrin (Covance PRB-145P, 1∶2000), K6 (Covance PRB-169P, 1∶2000) and fatty acid synthase (FASN)(Santa-Cruz SC-20140, 1∶1000).

### Oil Red O staining

Fresh unfixed epidermis sections were frozen on liquid nitrogen in Tissue-Tek® O.C.T (Sakura Finetek, #4583) compound and stored at −80°C. Frozen sections (10 microns) were washed successively in distilled water, 30% isopropanol, and 60% isopropanol. Slides were then incubated in Oil Red O for 15 minutes and subsequently washed in 60% isopropanol, 30% isopropanol, and water. Slides were counterstained with hematoxylin, rinsed in water for 10 minutes and coverslipped with glycerin jelly.

### Co-immunofluorescence staining

Tissue slides were deparaffinized and hydrated in successive washes (Xylene, 95% ethanol, 70% ethanol, PBS), blocked (K5,K10: 1 h with 1.5% natural goat serum (NGS), 1.5% natural horse serum (NHS), 1% bovine serum albumin (BSA) in PBS. C/EBPα,C/EBPβ: 2 h with 6%NGS, 6%NHS, 1%TritonX in PBS), and incubated overnight at 4°C with primary antibodies: K5 (rabbit polyclonal, Covance, 1∶1000), K10 (mouse monoclonal, Covance, 1∶1000), C/EBPα (rabbit polyclonal, Santa-Cruz SC-61, 1∶400), and C/EBPβ (mouse monoclonal, Santa-Cruz SC-7962, 1∶200). Slides were washed in PBS and incubated 45 minutes with fluorophore-linked secondary antibodies (Alexa Fluor® 568 goat anti-mouse (1∶1000), Alexa Fluor® 488 goat anti-rabbit (1∶1000)) in PBS, dehydrated and coverslipped with Dako Faramount Aqueous Mounting Medium.

### RNA and quantitative PCR

In brief, mice were killed by cervical dislocation and skin removed. Whole skin was minced in Sigma Tri-Reagent®, T-2494, sonicated on ice 4×10 s, centrifuged 14,000 g/10 m/4°C, and RNA was extracted with chloroform. Pelleted RNA was solubilized in water and then purified with DNase on Qiagen RNeasy® columns. cDNA was created with equal amounts of starting template with reverse transcriptase with appropriate no-RT controls. cDNA was then subjected to Real Time TaqMan® PCR (Applied Biosystems: keratin 10 (Mm03009921_m1), SCD3 (Mm00470480_m1), MC5R (Mm00442970_m1)) using GAPDH (Mm99999915_g1) as a housekeeping gene) and semi-quantitative PCR (all others) using the primers given in Table S1. For semi-quantitative PCR, PCR product was then run on 2% agarose gels with .01% ethidium bromide. After confirming PCR amplification was in the linear range by varying the amount of starting template, optical density of bands were determined by ImageJ™. Each experiment included 3 or more animals of each genotype.

## Results

### C/EBPα and C/EBPβ are co-expressed in skin

**Figure 1:**
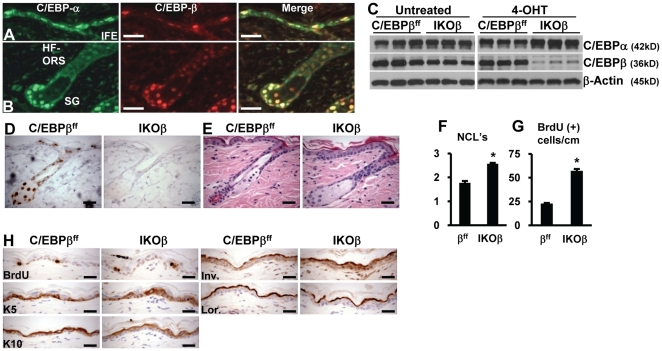
C/EBPα/β are co-expressed in skin and acute ablation C/EBPβ does not produce a major skin phenotype. Co-IF staining of untreated mouse skin in: (A) interfollicular epidermis (IFE), and (B) the hair follicle outer root sheath (HF-ORS) and sebaceous gland (SG). (C) Immunoblot analysis of epidermal lysates from untreated and 4OHT-treated C/EBPβ^ff^ and IKOβ mice (n = 3 mice/genotype/treatment). (D) IHC staining for C/EBPβ in 4OHT-treated C/EBPβ^ff^ and 4OHT-treated IKOβ mouse skin. (E) H&E staining of 4OHT-treated C/EBPβ^ff^ and 4OHT-treated IKOβ mouse skin. (F) Quantification of nucleated cell layers in 4OHT-treated C/EBPβ^ff^ and IKOβ mice (n = 3 mice/genotype/treatment). (G) Quantification of basal BrdU positive cells in 4OHT-treated C/EBPβ^ff^ and IKOβ mice (n = 3 mice/genotype/treatment). (H) IHC staining for BrdU and markers of squamous differentiation. All scale bars represent 30 microns. *indicates significantly different from controls p<.05, Student's t test.

The bZIP transcription factors, C/EBPα and C/EBPβ are expressed in mouse epidermis and sebaceous glands [23], [24], [34]. To determine whether C/EBPα and C/EBPβ are co-expressed within the same cell and not just within the same tissue or gland we performed co-immunofluorescence labeling for C/EBPα and C/EBPβ in untreated animals. As shown in Fig. 1A, C/EBPα and C/EBPβ are co-expressed in the nuclei of many interfollicular epidermal (IFE) keratinocytes. Most IFE suprabasal and basal keratinocytes that expressed C/EBPβ also expressed C/EBPα. However, some basal keratinocytes predominately expressed C/EBPα. The majority of outer root sheath (ORS) follicular keratinocytes co-expressed C/EBPα and C/EBPβ in their nuclei as did the vast majority of sebocytes of the sebaceous gland which displayed the highest levels of staining intensity for C/EBPα and C/EBPβ (Fig. 1B).

### Acute ablation of either C/EBPα or C/EBPβ alone in adult mouse skin does not produce a major skin phenotype

To ablate C/EBPα and C/EBPβ in postnatal adult mouse skin we used a conditional inducible approach utilizing K14-CreER^tam^ mice [36]. The K14 promoter directs the expression CreER^tam^ to the basal layer of the epidermis, ORS follicular keratinocytes of the hair follicle as well as the progenitor and proliferative populations of sebocytes of the sebaceous gland of mouse skin. The activity of CreER^tam^ is regulated through topical treatment with the synthetic ER antagonist, 4-hydroxytamoxifen (4OHT). While the overall goal was to simultaneously acutely ablate C/EBPα and C/EBPβ in adult mouse skin, it was first necessary to characterize the epidermis of adult mice in which C/EBPα or C/EBPβ were individually acutely ablated. While the phenotypes of the epidermal specific germline knockouts of C/EBPα and C/EBPβ have been previously characterized [25], [26], it could not be assumed that acute ablation in fully-developed adult skin would mimic the germline embryonic knockouts where developmental compensatory events could have occurred in the embryo to mask a postnatal phenotype. Moreover, in order to accurately define and interpret the phenotype of the double knockout it was important to determine the phenotype of single C/EBP knockouts in the same experimental model system.

K14-CreER^tam^;C/EBPα^ff^ mice and K14-CreER^tam^;C/EBPβ^ff^ mice were generated and we first analyzed K14-CreER^tam^;C/EBPβ^ff^ mice (hereafter referred to as (inducible knock out) IKOβ mice). Before treating mice with 4OHT, the levels of C/EBPβ protein in the epidermal homogenates of untreated IKOβ and untreated C/EBPβ^ff^ mice (β^ff^) were examined. Immunoblot analysis demonstrated epidermal C/EBPβ levels were similar in the two genotypes indicating the CreER^tam^ fusion protein was not significantly active in the absence of 4OHT treatment (Fig. 1C). 4OHT treatment (1.0 mg 4OHT/day for days 1–5 and 13–17; skin was collected on day 21), had no significant effect on C/EBPα or C/EBPβ levels in floxed-β mice (Fig. 1C), however, 4OHT treatment resulted in near complete ablation of C/EBPβ in IKOβ epidermis and this was consistently accompanied by a modest increase in C/EBPα levels suggesting possible compensatory upregulation (Fig. 1C). C/EBPβ is expressed in three forms (LAP* (39 kD), LAP (36 kD) and LIP (20 kDa) due to the use of alternative translation initiation sites and all forms of C/EBPβ were decreased in 4OHT-treated IKOβ mice (data not shown). Immunohistochemical (IHC) staining for C/EBPβ confirmed the loss of C/EBPβ in the IFE and revealed the loss of C/EBPβ in the ORS follicular keratinocytes of the hair follicle and sebocytes of sebaceous gland (Fig. 1D). The 4OHT-treated IKOβ epidermis displayed a moderate increase in: epidermal thickness (Fig. 1E right), the number of nucleated cell layers (Fig. 1F) and the number of BrdU positive basal keratinocytes following a 1 hour pulse was increased (Fig. 1G first panel). Overall, with the exception of the epidermal thickness and the increase in nucleated cell layers, the morphology of the IFE, ORS of the hair follicle and sebaceous glands of the 4OHT-treated IKOβ epidermis were indistinguishable from 4OHT-treated β^ff^ mice (Fig. 1E). IHC staining for K5, K10, loricrin and involucrin in C/EBPβ^ff^ and IKOβ epidermis treated with 4OHT revealed the correct spatial expression patterns, however, similar to germline C/EBPβ knockout mice [26], we observed a modest decreased staining intensity for K10 (Fig. 1H).

**Figure 2:**
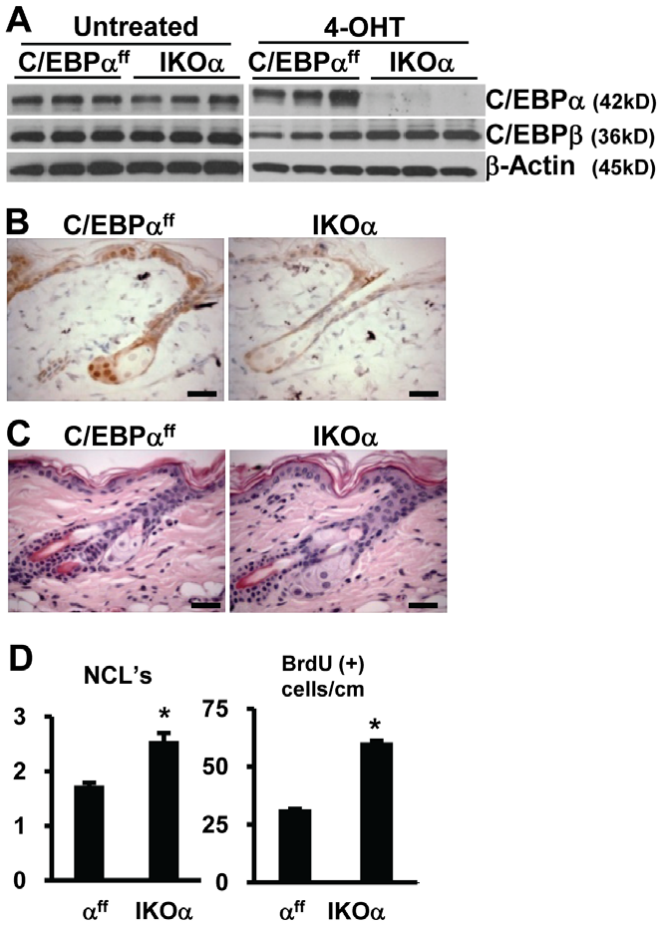
Acute ablation C/EBPα does not produce a major skin phenotype. (A) Immunoblot analysis of epidermal lysates from untreated and 4OHT-treated C/EBPα^ff^ and IKOα mice (n = 3 mice/genotype/treatment). (B) IHC staining for C/EBPα in 4OHT-treated C/EBPα^ff^ and 4OHT-treated IKOα mouse skin. (C) H&E staining of 4OHT-treated C/EBPα^ff^ and 4OHT-treated IKOα mouse skin. (D) Quantification of nucleated cell layers and number of basal BrdU positive cells in 4OHT-treated C/EBPα^ff^ and IKOα mice (n = 3 mice/genotype/treatment). All scale bars represent 30 microns. *indicates significantly different from controls p<.05, Student's t test.

Next, K14-CreER^tam^;C/EBPα^ff^ (IKOα) were examined. Baseline levels of C/EBPα protein in the epidermis of untreated IKOα mice and control C/EBPα^ff^ (α^ff^) mice were similar and the topical application of 4OHT to IKOα mice resulted in greatly reduced levels of C/EBPα and a modest increase in C/EBPβ (Fig. 2A). Both isoforms of C/EBPα ((p42 (42 kD) and p30 (30 kD)) were similarly decreased after treatment with 4OHT (data not shown). IHC staining for C/EBPα demonstrated the loss of C/EBPα in the IFE, ORS cells of the hair follicle and sebocytes in 4OHT treated IKOα animals (Fig. 2B). Epidermal thickness and basal cell proliferation as measured by BrdU incorporation was moderately increased in 4OHT-treated IKOα vs. 4OHT-treated C/EBPα^ff^ (Fig. 2D). With the exception of a slight increase epidermal thickness proliferation, the morphology of the IFE, ORS follicular epidermis and sebaceous glands of the 4OHT-treated IKOα epidermis were indistinguishable from 4OHT-treated C/EBPα^ff^ mice (Fig. 2C) as was the IHC staining for K5, K10, loricrin and involucrin in epidermis (data not shown). In summary, with the exception of modest increases in epidermal proliferation in 4OHT-treated IKOα mice, the acute single ablation of C/EBPα or C/EBPβ in adult mouse epidermis results in a phenotype similar to the germline knockout phenotype [25], [26], indicating developmental compensatory events do not have a major role in masking the postnatal germline knockout phenotype.

### Acute co-ablation of C/EBPα and C/EBPβ results in severe morphological defects in epidermis involving hyperplasia, dysplasia and hyperkeratosis

**Figure 3:**
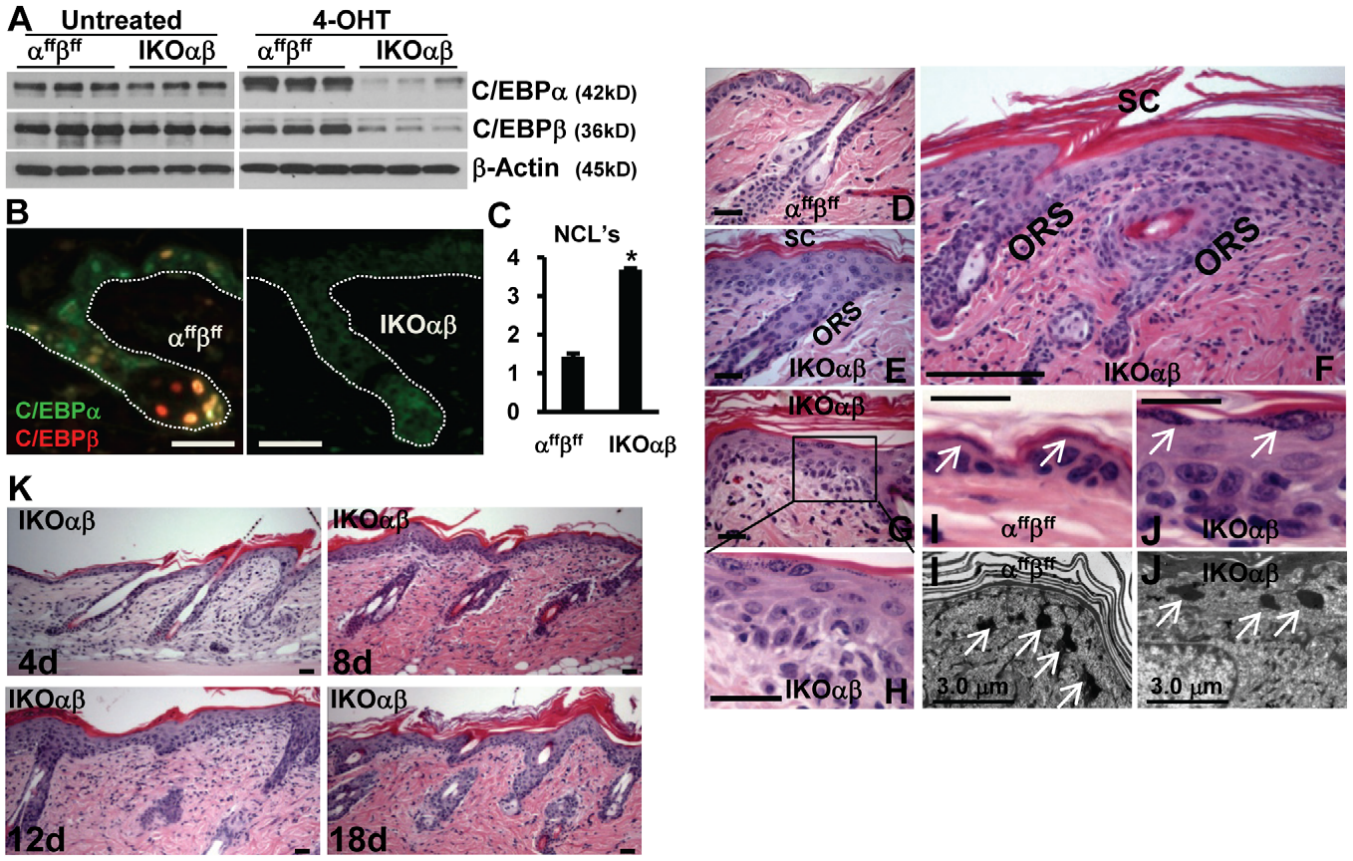
Acute co-ablation of C/EBPα/β results in epidermal morphological defects. (A) Immunoblot analysis of epidermal lysates from untreated and 4OHT-treated α^ff^β^ff^ and IKOαβ mice (n = 3 mice/genotype/treatment). (B) Co-IF staining for C/EBPα and C/EBPβ in 4OHT-treated α^ff^β^ff^ and 4OHT-treated IKOαβ mouse skin. (C) Quantification of epidermal nucleated cell layers in 4OHT-treated mice. (D) H&E staining of 4OHT-treated α^ff^β^ff^ mice. (E) H&E staining of 4OHT-treated IKOαβ mice. (F) Characteristic H&E staining of lesion in 4OHT-treated IKOαβ mouse. (G) H&E staining of 4OHT-treated IKOαβ epidermis with (H) region enlarged displaying dysplasia. (I) H&E staining and TEM micrograph of keratohyalin granules in 4OHT-treated (I) α^ff^β^ff^ and 4OHT-treated (J) IKOαβ epidermis. (K) H&E staining of lesions from 4OHT-treated animals from different days after start of 4OHT treatment. Scale bars represent 30 microns unless otherwise notated. *indicates significantly different from controls p<.05, Student's t test.

To address potential functional redundancies between C/EBPα and C/EBPβ and unmask their function in epidermis and sebaceous glands, K14-CreER^tam^;C/EBPα^ff^;C/EBPβ^ff^ (IKOαβ) and C/EBPα^ff^;C/EBPβ^ff^ (α^ff^β^ff^) mice were generated and characterized. Immunoblot analysis demonstrated the levels of C/EBPα and C/EBPβ in the untreated epidermis of IKOαβ mice and α^ff^β^ff^ mice were similar (Fig. 3A), and the topical application of 4OHT to IKOαβ and α^ff^β^ff^ mice resulted in dramatic decreases in both C/EBPα and C/EBPβ in IKOαβ but not 4OHT-treated α^ff^β^ff^ mice (Fig. 3A). All forms of C/EBPβ (LAP*, LAP and LIP) and C/EBPα (p42 and p30) were similarly decreased (data not shown). Loss of C/EBPα and C/EBPβ in the IFE, ORS follicular keratinocytes and sebocytes was demonstrated by co-IF staining for C/EBPα and C/EBPβ in 4OHT-treated IKOαβ and 4OHT-treated α^ff^β^ff^ mouse skin (Fig. 3B). In normal IFE, basal keratinocytes progress upwards through morphologically distinct spinous and then granular suprabasal layers, eventually ending in the production of a nonviable stratum corneum or cornified layer. Histological analysis of dorsal skin sections from 4OHT-treated IKOαβ and 4OHT-treated α^ff^β^ff^ mice revealed α^ff^β^ff^ IFE was normal (Fig. 3D). In contrast, the 4OHT-treated IKOαβ IFE and infundibular ORS keratinocytes were hyperplastic (Fig. 3E&F) and the IFE displayed a significant increase in the number of nucleated epidermal cell layers (Fig. 3C). In addition, the basal and spinous layers of the 4OHT-treated IKOαβ epidermis were dysplastic as characterized by the disorganization of the layers and by highly abnormal variations in cell and nucleus size (Fig. 3G&H). There were also focal and regional areas of moderate to severe hyperplasia of the follicular infundibular epithelium and ORS keratinocytes (Fig. 3F) in the 4OHT-treated IKOαβ epidermis. In contrast to the disorganized basal and spinous layers, the granular layer of keratinocytes in 4OHT-treated IKOαβ mice appeared less affected and keratohyalin granules, a hallmark of granular layer, were present at levels similar to 4OHT-treated control mice (Fig. 3I-J). The stratum corneum in 4OHT-treated IKOαβ epidermis was significantly thickened (hyperkeratosis) (Fig. 3E-G) and displayed low levels of parakeratosis. Collectively, these morphological and histological alterations in the 4OHT-treated IKOαβ epidermis indicate the acute loss of C/EBPα and C/EBPβ disrupts epidermal homeostasis, and within the viable epidermis, the basal and spinous layers appear to be most severely affected. We also examined mice at different times after the start of 4OHT treatment to determine when epidermal abnormalities appeared. We observed focal areas of hyperplasia in as early as four days from start of treatment and these areas became more extensive and severe at 8, 12, and 18 days from start of treatment (Fig. 3K).

### Acute co-ablation of C/EBPα and C/EBPβ results in specific molecular defects in squamous differentiation

**Figure 4:**
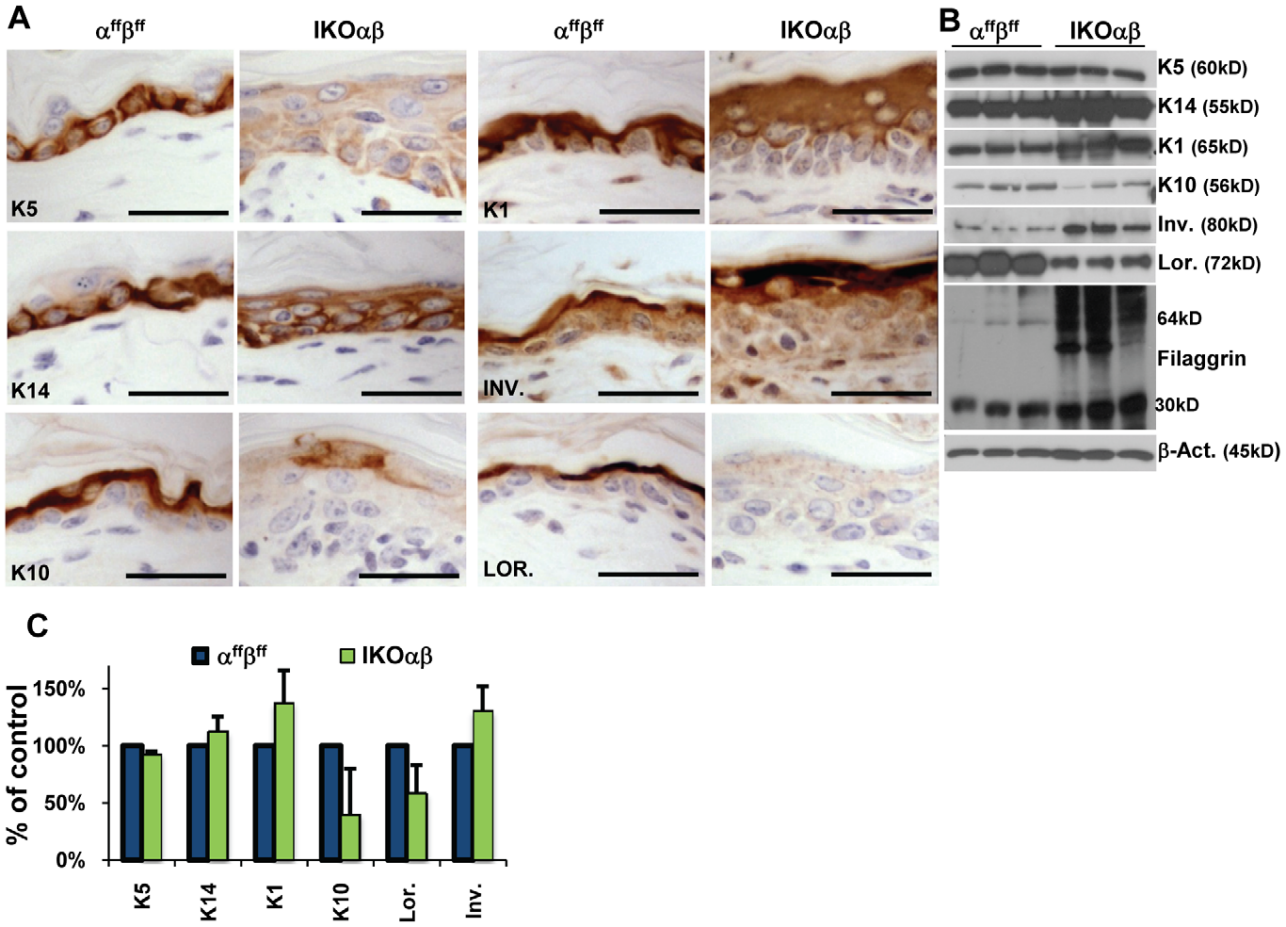
Co-ablation of C/EBPα/β results in defects in epidermal squamous differentiation. (A) IHC staining for markers of stratified squamous differentiation in 4OHT-treated controls and 4OHT-treated inducible double knockouts (IKOαβ). (B) Immunoblot analysis of epidermal lysates from animals treated with 4OHT for markers of stratified squamous differentiation (n = 3 mice/genotype). (C) Taqman® real time quantitative PCR (K10) and reverse transcriptase semi-quantitative PCR (all others) on mRNA from 4OHT-treated whole skin (n = 5 mice/genotype). Scale bars represent 30 microns.

4OHT-treated IKOαβ epidermis was analyzed for specific molecular defects in differentiation using IHC staining for molecular markers of specific stages of squamous differentiation. IHC staining in α^ff^β^ff^ and IKOαβ epidermis from mice treated with 4OHT were conducted side by side and 3,3′-diaminobenzidine (DAB) incubation time was standardized to 4OHT-treated α^ff^β^ff^ epidermis. In 4OHT-treated IKOαβ epidermis, the basal cell markers, K5 and K14, were no longer restricted to the basal layer, but were aberrantly expressed throughout all suprabasal layers of the epidermis (Fig. 4A). However, K14 and especially K5 IHC staining were significantly less intense in 4OHT-treated IKOαβ epidermis than in 4OHT-treated α^ff^β^ff^ epidermis. Immunoblot analysis of epidermal lysates from 4OHT treated α^ff^β^ff^ and 4OHT-treated IKOαβ epidermis (Fig. 4B) revealed increased levels of K14 in IKOαβ epidermis, while K5 showed similar levels to α^ff^β^ff^ controls substantiating the IHC results showing reduced levels but a greatly expanded K5 compartment (Fig. 4A). K10, an early marker of the spinous layer and of the basal to spinous transition was significantly reduced in 4OHT-treated IKOαβ epidermis (Fig. 4A). K1, another early marker of the spinous layer was delayed in its expression in 4OHT-treated IKOαβ epidermis and instead of being expressed in the layer adjacent to the basal cell layer, K1 was not expressed until ∼2-3 layers of cells above the basal layers (Fig. 4A). Immunoblot analysis for K1 and K10 confirmed the IHC results (Fig. 4B). In general, markers of later stages of differentiation such as involucrin and filaggrin were not decreased. Involucrin, a component of the cornified envelope and a marker of the granular layer, was expressed in the appropriate layers but at higher levels compared to similarly treated α^ff^β^ff^ epidermis (Fig. 4A-C). Filaggrin, a marker of the granular layer was also expressed at higher levels in 4OHT-treated IKOαβ epidermis than in similarly treated α^ff^β^ff^ epidermis (Fig. 4B). In contrast, the expression of loricrin, a component of the cornified envelope, was significantly diminished in 4OHT-treated IKOαβ epidermis (Fig. 4A-C). In general, mRNA levels of the various differentiation markers were in accord with immunoblot and IHC levels (Fig. 4C). Overall the molecular changes in K5, K14, K1, and K10 in the 4OHT-treated IKOαβ epidermis are consistent with a defect in the basal to spinous transition.

### Acute co-ablation of C/EBPα and C/EBPβ results in hyperproliferative basal and suprabasal keratinocytes

**Figure 5:**
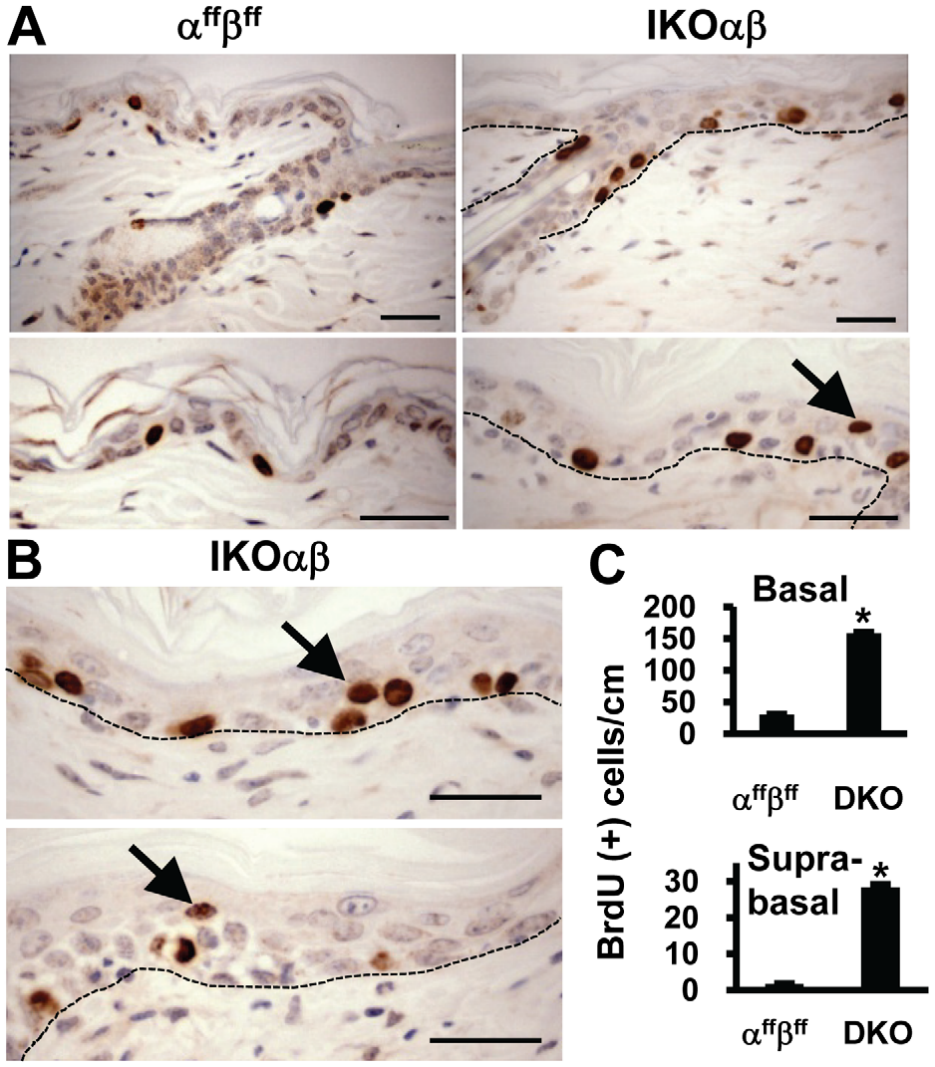
Co-ablation of C/EBPα/β results in hyperproliferative basal and suprabasal keratinocytes. (A) IHC staining for BrdU-positive keratinocytes in HF-ORS and IFE in 4OHT-treated α^ff^β^ff^ and 4OHT-treated IKOαβ epidermis. (B) Suprabasal BrdU-positive S-phase keratinocytes in 4OHT-treated IKOαβ mice. (C) Quantification of BrdU-positive basal and suprabasal IFE keratinocytes in 4OHT-treated α^ff^β^ff^ and 4OHT-treated IKOαβ mice (n = 3 mice/genotype, 3 strips per mouse; *p<0.05). Scale bars represent 30 microns.

Cell proliferation analysis was conducted using a 1 h pulse label with BrdU, followed by IHC analysis for BrdU positive S-phase cells. 4OHT-treated IKOαβ epidermis demonstrated a significant increase in the numbers of BrdU positive S-phase cells in the IFE and ORS keratinocytes (Fig. 5 A&B) over 4OHT-treated α^ff^β^ff^ epidermis. In 4OHT-treated IKOαβ IFE, there was a 4.7 fold increase in BrdU positive basal S-phase keratinocytes compared to similarly treated α^ff^β^ff^ mice (Fig. 5C). In normal epidermis, suprabasal proliferating keratinocytes are infrequent as proliferating keratinocytes are restricted to the basal layer. Analysis of 4OHT-treated IKOαβ epidermis revealed suprabasal BrdU positive S-phase keratinocytes were greatly increased and were observed at a ∼27 fold increase over 4OHT-treated α^ff^β^ff^ epidermis (Fig. 5B&C). Collectively, these results demonstrate hyperproliferative basal and suprabasal keratinocytes and suggest an impairment of basal keratinocyte cell cycle withdrawal in mice missing C/EBPα and C/EBPβ.

### Acute co-ablation of C/EBPα and C/EBPβ results in molecular, morphological and differentiation defects in sebocytes

**Figure 6:**
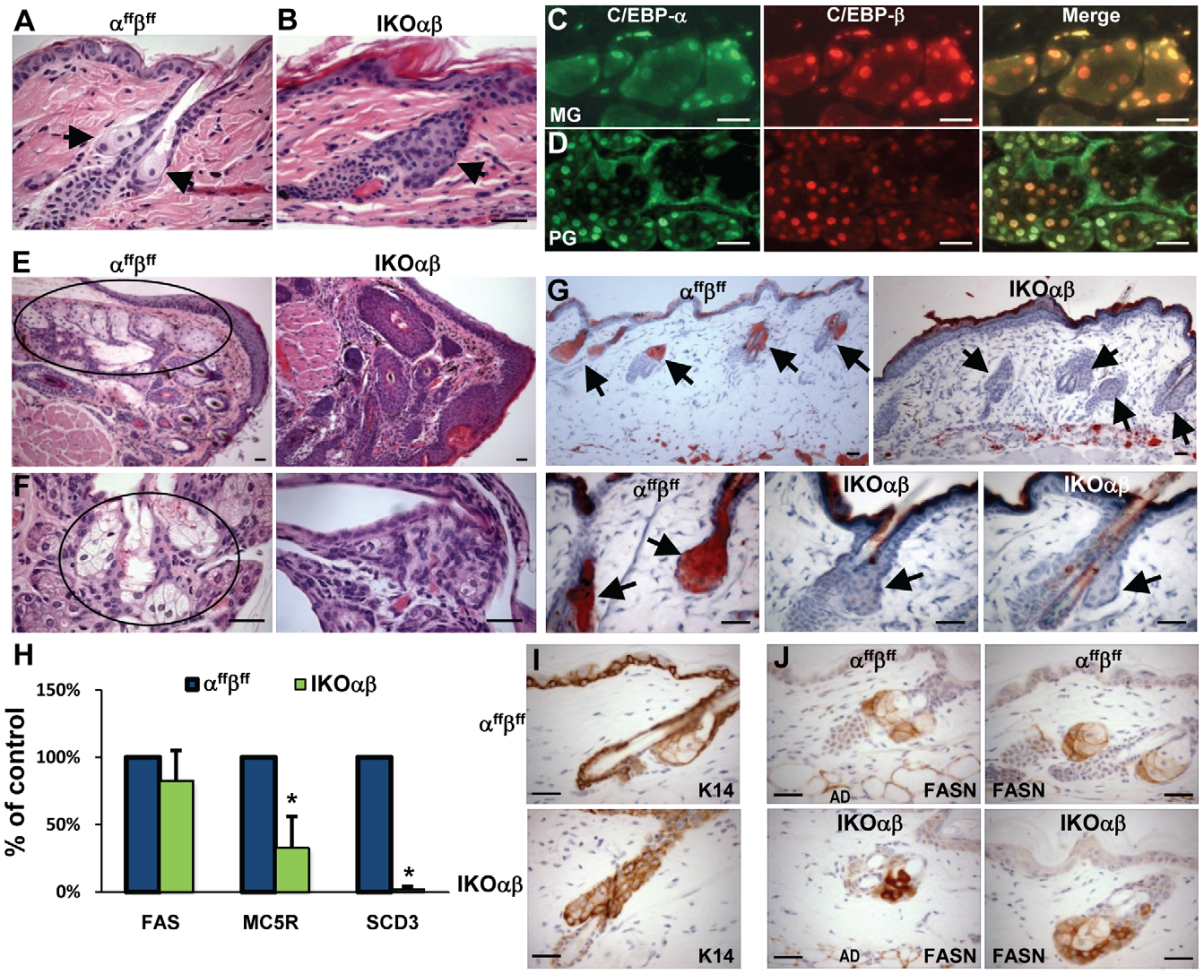
Co-ablation of C/EBPα/β results in morphological and molecular defects in sebaceous, Meibomian and preputial glands. (A) H&E staining of sebaceous glands/lobules of 4OHT-treated α^ff^β^ff^ and (B) 4OHT-treated IKOαβ mice. Co-IF staining in (C) Meibomian glands and (D) preputial glands in untreated controls. H&E staining of (E) Meibomian glands (circled) and (F) preputial glands (circled) of 4OHT-treated α^ff^β^ff^ and 4OHT-treated IKOαβ mice. (G) Oil Red O staining for lipids in 4OHT-treated α^ff^β^ff^ (arrows point to sebaceous glands) and 4OHT-treated IKOαβ mice (arrows point to sebaceous lobules). (H) FASN: Semi-quantitative reverse transcriptase PCR on whole skin mRNA from 4OHT-treated α^ff^β^ff^ and 4OHT-treated IKOαβ mice. SCD3 and MC5R: TaqMan® Real Time PCR. (n = 5 mice/treatment/genotype; *p<0.05). IHC staining for (I) keratin 14 and (J) FASN in 4OHT-treated α^ff^β^ff^ and 4OHT-treated IKOαβ mouse epidermis (AD-Adipose Tissue). Scale bars represent 30 microns.

In untreated mouse skin, sebaceous glands are associated with the upper follicle and these glands contained numerous sebocytes with a large light staining, finely vacuolated, clear translucent cytoplasm and a distinct round nucleus. Small reserve cells, (proliferative undifferentiated sebocytes) were less numerous and were peripheral in the glands (Fig. 6A). In the skin of 4OHT-treated IKOαβ mice, hair follicles contained distinct sebaceous gland lobules in the typical location on the hair follicle (Fig. 6B); however, these glands contained unusual looking sebocytes that appeared undifferentiated, had reduced cytoplasmic volume and lacked clear fine vacuolation supportive of lipid accumulation. Affected glands had an increased cellular density with more closely apposed nuclei and the cells were morphologically reminiscent of the reserve undifferentiated cells. To determine whether other sebaceous type glands were similarly affected, we examine the Meibomian and preputial glands for C/EBPα and C/EBPβ expression and for alterations in 4OHT-treated IKOαβ mice. Meibomian glands are found at the rim of the eyelid of mammals and the Meibomian sebocytes produce and release a sebum-like substance onto the eye that functions to prevent the evaporation of the tear film on the eye [40]. The preputial gland, found in some male mammals and located in the subcutaneous tissue of the inguinal area, is involved in the production and secretion of pheromones and lipids. Co-IF staining revealed C/EBPα and C/EBPβ were co-expressed in sebocytes of both the Meibomian and preputial glands (Fig. 6C&D). Gross examination of 4OHT-treated IKOαβ mice revealed that eyes of these mice appeared dry, swollen, and partially closed. Histological analysis revealed atrophied Meibomian glands characterized by greatly reduced lobule size and diminished numbers of differentiated sebocytes with clear vacuolated cytoplasm (Fig. 6E). In addition, there was severe regional epidermal hyperplasia of the haired skin eyelid epidermis as well as hyperplasia of the Meibomian ducts and follicular infundibula, which was similar to skin epithelium (Fig. 6E right panel). In contrast, eyes of 4OHT-treated α^ff^β^ff^ mice were normal and the mice contained normal appearing Meibomian glands and ducts (Fig. 6E). Preputial glands from 4OHT-treated IKOαβ mice were also abnormal. The 4OHT-treated α^ff^β^ff^ mice displayed preputial glandular cells with typical, abundant, clear, finely vacuolated, translucent cytoplasm and distinct round nuclei (Fig. 6F circle). In contrast, 4OHT-treated IKOαβ mice had marked atrophy of preputial gland lobules and decreased numbers of clear finely vacuolated sebocytes (Fig. 6F right). Thus, the acute lost of C/EBPα and C/EBPβ severely impairs sebaceous, Meibomian, and preputial gland homeostasis and results in the loss of morphologically distinct differentiated sebocytes in all of these glands. To provide biochemical evidence for altered sebocyte differentiation, histological sections of skin of 4OHT-treated α^ff^β^ff^ and 4OHT-treated IKOαβ mice were subjected to Oil Red O staining. Oil Red O stains lipid and sebum and positive Oil Red O staining of sebaceous glands is a hallmark of sebocyte differentiation. In 4OHT-treated α^ff^β^ff^ mice, Oil Red O stained all sebaceous glands, demonstrating the presence of terminally differentiated sebum producing sebocytes associated with nearly every hair follicle (Fig. 6G). In contrast, the sebaceous gland lobules associated with hair follicles in 4OHT-treated IKOαβ mice did not stain with Oil Red O (Fig. 6G right and bottom right panels) indicating the absence of sebum and terminally differentiated sebocytes. To further characterize the sebaceous gland defect in 4OHT-treated IKOαβ mice, we examined K14, fatty acid synthase (FASN), stearoyl-CoA desaturase (SCD3) and melanocortin 5 receptor (MC5R). K14 is expressed in the basal IFE keratinocytes and is also expressed in the undifferentiated peripheral sebocytes in sebaceous glands, while SCD3 and MC5R have been characterized as specific markers of sebocyte differentiation. Within the sebaceous glands of 4OHT-treated α^ff^β^ff^ mice, K14 is appropriately expressed in the undifferentiated sebocytes around the periphery of sebaceous gland (Fig. 6I). In contrast, the entire 4OHT-treated IKOαβ sebocyte lobule uniformly expressed K14 suggesting that the lobule may be composed of a population of undifferentiated sebocytes. To further identify these cells within the lobule as sebocytes we conducted IHC staining for FASN. Sebaceous glands in 4OHT-treated α^ff^β^ff^ mice expressed FASN as did 4OHT-treated IKOαβ sebocyte lobules indicating that the lobules are comprised of sebocytes (Fig. 6J). The intense FASN staining of 4OHT-treated IKOαβ sebocyte lobules compared to control glands is likely due to reduced cytoplasmic volume of IKOαβ sebocytes. FASN mRNA levels were similar in skin between the two genotypes (Fig. 6H). SCD3 and MC5R are markers of terminally differentiated sebocytes and real time quantitative PCR revealed mRNA of both were greatly reduced in 4OHT-treated IKOαβ. Collectively these morphological, biochemical and molecular results indicate that acute co-ablation of C/EBPα and C/EBPβ in adult mice results in a significant sebaceous gland defect in which sebocyte terminal differentiation and lipid production is impaired.

## Discussion

The co-ablation of C/EBPα and C/EBPβ in adult mouse skin revealed critical biological functions for these transcription factors in keratinocytes and sebocytes that are not present in germ line single knockout mice [25], [26] as well as the inducible conditional single knockout mice described in this study. For example, sebaceous glands of C/EBPα or C/EBPβ knockout mice were indistinguishable from wild type sebaceous glands. In contrast, the co-ablation of C/EBPα and C/EBPβ resulted in severe sebaceous gland morphological defects and a failure of sebocyte differentiation. Likewise, acute co-deletion of C/EBPα and C/EBPβ produces profound changes in epidermal homeostasis not seen in single KOs that involves the disruption of stratified squamous differentiation, disorganization of the epidermis and the hyperproliferation of the IFE keratinocytes as well as ORS follicular keratinocytes. Thus the co-ablation of C/EBPα and C/EBPβ has revealed previously unknown functional roles for these transcription factors in sebocyte biology and has confirmed and eclipsed earlier studies in C/EBPβ knockout mice that indicated a role for C/EBPβ in early events of keratinocyte differentiation involving K1/K10 expression and growth arrest [26]. Our results indicate that functional redundancies exist between C/EBPα and C/EBPβ and/or these two transcription factors cooperate to regulate basal keratinocyte cell cycle withdrawal and early differentiation in keratinocytes and sebocytes.

Sebaceous glands are formed from a sebocyte progenitor that expresses Blimp 1, keratin 14 (K14) and K5 [29], and these cells produce a proliferative population of sebocytes [30], [32] which in turn differentiate into the lipid/sebum producing cells. Through the inducible conditional co-ablation of C/EBPα and C/EBPβ we unmasked novel roles for these transcription factors in sebaceous gland differentiation. Sebocytes lacking C/EBPα and C/EBPβ were morphologically altered and sebum production was blocked. IKOαβ mice retained distinct sebaceous gland lobules, but these cells did not produce sebum, a hallmark of sebocyte differentiation. It appears the proliferating cells located on the periphery of the gland continued dividing but their progeny were unable to initiate terminal differentiation. Our observation that the cells in the lobules of IKOαβ mice express K14 and FASN is consistent with this idea, providing compelling evidence that the cells within the lobule are sebocytes. In addition, we observed decreased levels of SCD3 and MC5R, two markers of differentiated sebocytes. Our results indicate the adipogenic transcription factors, C/EBPα and C/EBPβ, also have critical roles in sebocyte differentiation through the regulation of lipogenesis and sebum production. The critical importance of these transcription factors in sebocyte homeostasis was further strengthened by the profound effect of their co-ablation on the specialized sebocytes of the Meibomian and preputial glands. Meibomian glands of mice lacking C/EBPα and C/EBPβ were severely disorganized, dysplastic and there was evidence of bacterial infection. Posterior blepharitus is a human disease often characterized by improper sebum production by Meibomian glands, lending an environment favorable for bacterial growth, and accompanied by inflammation [41]. Acne, seborrheic dermatitis, blepharitis, and sebaceous cancer are among several human health conditions involving altered sebocyte homeostasis. It seems plausible the transcription factors C/EBPα and C/EBPβ could have involvement in these disease conditions.

Molecular analysis of IKOαβ epidermis revealed that K5 and K14, markers of basal keratinocytes, were expressed throughout the basal and suprabasal layers, while suprabasal markers K1 and K10 were delayed or weakly expressed. A concomitant increase in hyperproliferative basal and suprabasal IFE keratinocytes was observed in IKOαβ epidermis. These results indicate an expansion of the basal compartment and reduced ability of these basal keratinocytes to initiate the early events of stratified squamous differentiation involving K1 and K10 expression and cell cycle withdrawal. Both C/EBPα and C/EBPβ can bind to and activate the K10 promoter and C/EBP binding sites [42] have been identified in both the K1 and K10 promoters [26] suggesting differences in K1 and K10 expression could be regulated directly by C/EBPs at the promoter level. Chromatin immuno-precipitation experiments will be required to resolve these issues.

In general, the granular layers of IKOαβ epidermis appeared less affected; keratohyalin granules were present and involucrin and filaggrin were expressed, albeit at increased levels. These changes in increased involucrin and filaggrin expression could be a response to the disruption of epidermal homeostasis due to the defective basal to spinous transition or it is possible that C/EBPα and C/EBPβ contribute to the suppression of granular differentiation as has been reported for Hes1 in epidermal development [19].

Mechanisms of epidermal development and postnatal differentiation are not necessarily synonymous. For example, ablation of Notch in epidermal development produces the loss of spinous and granular layers and a hypoproliferative phenotype [18], while postnatal ablation of Notch1 produce an opposite effect involving a hyperproliferative phenotype and an increased granular layer [20]. Hes1 ablation dramatically alters epidermal development involving the spinous to granular transition, but appears to have a minimal role in adult skin [19]. Recently the co-deletion of C/EBPα and C/EBPβ in developing mouse skin produced mice that died soon after birth, and the analysis of epidermis from these mice revealed hyperplasia, decreased expression of spinous and granular markers of differentiation and a role for E2F in the hyperplastic epidermis [28]. While we observed similar changes in basal cell hyperproliferation and defects in spinous markers of differentiation, we did not observe the granular changes reported in developing skin. For example, we did not observe diminished keratohyalin granules, filaggrin, and involucrin expression in adult epidermis and we did observe an uncoupling of involucrin and loricrin expression in adult epidermis. Collectively, these results indicate that there are significant differences between the effects of co-deletion of C/EBPα and C/EBPβ in embryonic development and postnatal differentiation.

Notch signaling is important in both the early and later stages of epidermal development where it regulates the basal to spinous transition in a Hes1 independent manner and the spinous to granular transition in a Hes1 dependent manner [18]–[20]. AP-2α and AP-2γ also have essential roles in both the early and later stages of epidermal development and recently it was reported that both AP-2 and Notch signaling converge on the regulation of the expression of C/EBPα and C/EBPβ [21]. Results reported in our current study demonstrate C/EBPα and C/EBPβ have critical roles in the early stages of squamous differentiation and our results are consistent with a model where C/EBPs function downstream of AP-2 and Notch signaling to regulate the basal to spinous transition.

As described in the introduction, the knockin of C/EBPβ into the C/EBPα locus rescues the lethality of C/EBPα^−/−^ mice and supports the notion of functional redundancies between these family members [27], [43]. However, not all of the phenotypes of C/EBPα^−/−^ mouse were reversed suggesting that there are also unique C/EBP family member functions. Likewise our current study provides evidence for functional redundancies between C/EBPα and C/EBPβ in epidermal and sebocyte homeostasis. However, in terms of skin tumorigenesis, these family members have unique functions. For example, C/EBPα is a skin tumor suppressor [25] and a regulator of the G1 checkpoint in keratinocytes in the DNA damage response [44]. In contrast, C/EBPβ is a mediator of keratinocyte survival and is required for skin tumorigenesis [45]. Understanding the redundant and unique roles these two transcription factors have will be important in the eventual elucidation of their roles in tumorigenesis.

## Supporting Information

Table S1 Primers used for semi-quantitative PCR.
(0.03 MB DOC)
