# Supplementary material for: C/EBPα and C/EBPβ Are Required for Sebocyte Differentiation and Stratified Squamous Differentiation in Adult Mouse Skin
Source: PLoS One. 2010 Mar 23;5(3):e9837. doi: 10.1371/journal.pone.0009837 (PMC2843749; doi:10.1371/journal.pone.0009837)
Supplement: Table S1 — Primers used for semi-quantitative PCR. (0.03 MB DOC) [file pone.0009837.s001.doc]

**Table S1. Primers used for semi-quantitative PCR**

| **Gene** | **5’-3’ Forward Primer** | **5’-3’ Reverse Primer** |
| --- | --- | --- |
| **FASN** | AGCGGCCATTTCCATTGCCC | CCATGCCCAGAGGGTGGTTG |
| **Keratin 1** | GACACCACAACCCGGACCCAAAACTTAGAC | ATACTGGGCCTTGACTTCCGAGATGATG |
| **Keratin 5** | AACCTCCAGAACGCCATTGC | GCCAGAAGAGACACTGTTTGTAACG |
| **Keratin 14** | TTGGTAGTGGATTTGGTGGTCG | GATGGTCTTGAAGTAGGGGCTGTAG |
| **Loricrin** | TCCTCTCAGCAGACCAGTCAG | GGTAGTCATTCAGAAACCAAGATG |
| **Involucrin** | GAGCGTGAAGGTTATCAAGGACC | GGTGTGGTTGCTTTAGTTTTGGC |
| **GAPDH** | GAAGGTCGCTGTGAACGGA | GTTAGTGGGGTCTCGCTCCT |
